# Sustainable Pultruded Sandwich Profiles with Mycelium Core

**DOI:** 10.3390/polym15153205

**Published:** 2023-07-28

**Authors:** Marion Früchtl, Andreas Senz, Steffen Sydow, Jonas Benjamin Frank, Andrea Hohmann, Stefan Albrecht, Matthias Fischer, Maximilian Holland, Frederik Wilhelm, Henrik-Alexander Christ

**Affiliations:** 1 Fraunhofer Institute for Casting, Composite and Processing Technology IGCV, Am Technologiezentrum 2, 86159 Augsburg, Germany; 2 Fraunhofer Institute for Wood Research Wilhelm-Klauditz-Institut WKI, Riedenkamp 3, 38108 Braunschweig, Germany; 3 Fraunhofer Institute for Building Physics IBP, Nobelstraße 12, 70569 Stuttgart, Germany

**Keywords:** sustainable composite, pultrusion, mycelium, life cycle assessment, sandwich, hybrid pultruded products, bio-based, sustainable GFRP sandwich

## Abstract

This research focuses on exploring the potential of mycelium as a sustainable alternative to wood or solid foam in pultruded glass fiber-reinforced plastic (GFRP) sandwich profiles. The study evaluates the performance and the environmental sustainability potential of this composite by mechanical tests and life cycle assessment (LCA). Analysis and comparison of pultruded sandwich profiles with mycelium, polyurethane (PUR) foam and chipboard demonstrate that mycelium is competitive in terms of its performance and environmental impact. The LCA indicates that 88% of greenhouse gas emissions are attributed to mycelium production, with the heat pressing (laboratory scale) being the main culprit. When pultruded profiles with mycelium cores of densities 350 and 550 kg/m³ are produced using an oil-heated lab press, a global warming potential (GWP) of 5.74 and 9.10 kg CO_2_-eq. per functional unit was calculated, respectively. When using an electrically heated press, the GWP decreases to 1.50 and 1.78 kg CO_2_-eq. Compared to PUR foam, a reduction of 23% in GWP is possible. In order to leverage this potential, the material performance and the reproducibility of the properties must be further increased. Additionally, an adjustment of the manufacturing process with in situ mycelium deactivation during pultrusion could further reduce the energy consumption.

## 1. Introduction

Fungi are natural examples of the recycling and utilization of organic waste materials. With their network of hyphae, they grow through a substrate and thus form the fungal mycelium [1]. The mycelium acts as a natural binder and creates a stable and at the same time biodegradable composite material [2]. This material is typically based on organic residues from industry or agriculture, e.g., hemp hurds or wood chips, and could hence be usefully employed in a future bio-based circular economy [3]. Complex geometries can already be achieved in the production process and concretized by subsequent steps, such as hot board pressing. The achievable material properties of the mycelial material depend on the respective combination of substrate and fungal spores used [4,5]. Compared to commercial wood or foam materials, however, the mechanical performance of the mycelial material is limited in terms of the mechanical stability and also highly dependent on the substrate [6]. While hot pressing already significantly increases the mechanical stability, other post-processing methods are conceivable [7]. 

Mycelium materials have great potential to replace, e.g., conventional fossil-based sandwich materials in various manufacturing processes, such as pultrusion, and serve different industries, thereby contributing to a more sustainable economy [3,8,9,10]. 

Pultrusion is an efficient, highly automated and continuous manufacturing process with high production and relatively low material waste rates, used to produce fiber-reinforced composite materials with constant cross-section and high fiber content, resulting in excellent mechanical and lightweight properties [11,12]. Pultrusion involves pulling continuous fibers (e.g., glass or carbon fiber) and textiles through a resin bath or impregnation chamber to impregnate them, usually with thermosets, such as polyester or epoxy. The aligned fibers are then pulled through a heated die or mold initiating the curing process and determining the final shape of the profile [13,14]. The wide application range of pultruded profiles extends from medical and dental applications [15,16,17] over transport and automotive sectors [18,19], energy, infrastructure and construction [20,21,22]. The pultrusion process also enables the manufacturing of sandwich structures in order to improve product properties such as strength-to-weight ratio, thermal or acoustic insulation and moment of inertia that are required in specific fields of application [23,24]. Within pultrusion, the sandwich core itself could either be produced in-line in the case of polyurethane (PUR) foam [25] and phenolic foam [26] or used as an already manufactured preform for materials such as cork, extruded polystyrene, PUR or wood [23,24,27,28]. 

The use of mycelium in pultruded sandwich profiles represents a promising approach to replace traditional fossil core materials with sustainable alternatives, while further improving the mechanical properties of the material. 

Provided that comparable properties can be achieved, studies indicate that it should be possible to substitute fossil-based cores with pressed mycelial materials, where the density can be affected by the pressing parameters [5,29]. In addition, the exceptional ability of fungi to recycle organically, and to grow under non-critical parameters (without pesticides), make it an interesting material for pultruded components and related markets [30]. This bears great potential to significantly contribute to the national bio-economy strategy [31] as well as to the global sustainable development goals [32]. In this context, particularly the utilization of agricultural waste for the mycelium growing process and its ability for carbon dioxide sequestration should be mentioned. In particular, bio-composites, biopolymers and natural fiber composites (NFC) have the potential to replace fossil-based plastics. In the process, conventional methods in numerous industrial sectors are being challenged to enable a successful transition from a linear to a circular economic model, which is mainly characterized by resource conservation as well as waste reduction. This also requires, next to an increasing use of renewable raw materials, innovative end of life strategies that allow for the preservation of materials in the economic cycle and thus preserve value creation. 

According to recent studies, bio-composites made from mycelium have been demonstrated to be an alternative option for the production of various materials used in the construction industry, in design materials or in the food industry in accordance with other materials derived from biological processes, such as plant extracts or agricultural residues [8,9,33]. However, despite the large potential the pultrusion of mycelium sandwich cores can provide, there are not many research studies on this topic [34,35]. 

This work provides first insights into the potential of mycelium for pultruded sandwich material from a performance and environmental point of view. For this purpose, in the following, the process route and the boundary conditions for the evaluation are described. An analysis regarding the material performance and environmental impact are presented comparing mycelium with PUR foam and chipboard as core materials. This first investigation on the combination of mycelium and pultrusion could be a possible sustainable solution to substitute traditional sandwich cores made of hard foam in many applications, when raised from a laboratory scale to an industrial level. 

## 2. Materials and Methods

### 2.1. Mycelium Cultivation and Sample Preparation

**Figure 1:**
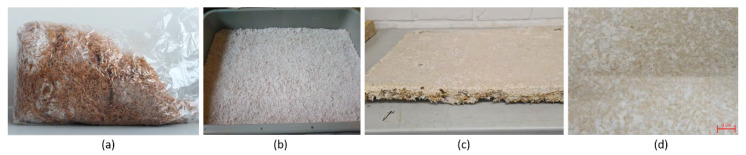
Mycelium cultivation and growth. (**a**) Inoculation of substrate using mycelium in growth bag; (**b**) growth of the material in a polypropylene (PP) growth box; (**c**) living mycelium after approximately 14 days of growth. (**d**) Zoom of the mature mycelium and the growth structure.

For the production of the mycelial material, hemp hurds pre-inoculated with the fungus Ganoderma spp. were used and initially stimulated to grow by adding water (50 mL) and flour (25 g/kg), as described below. Hemp hurds were selected as the standard substrate, as these had demonstrated both a controllable growth process and good mechanical parameters after the pressing process in previous trials. Conventional polypropylene (PP) boxes with lids (60 × 40 cm²) functioned as cultivation boxes, which were filled to approximately 2.5 to 3 cm with the mycelium/substrate mixture. The growth process was carried out in a standard climate with 65% humidity at 20 °C, without light. The growth time varied between ten and 14 days. Before the fruiting body formation started, growth was interrupted by a subsequent pressing step, as described in Table 1. Furthermore, Figure 1 shows the first steps from the mycelium cultivation to the growth of the plates. Clearly visible are the overgrown structures by the mycelium. In this process, the fungus digests only part of the substrate and grows mainly around it. The fact that the majority of the original substrate remains in place enables it to provide additional stability to the system in the subsequent pressing process. 

Deactivating the mycelial material to avoid the uncontrolled fruiting body formation and further growth is typically achieved through heat treatment in a drying oven in commercial settings (70 to 80 °C for 2 h). For the production of mycelium-preforms for pultrusion, a pressing step is necessary which can be combined with the mycelium deactivating step directly, as both steps require temperatures from 160 to 200 °C. Consequently, the mycelium is inactivated while being pressed and formed at the same time. For this study, two target densities for the mycelium-preforms were defined. Table 1 lists the different parameter sets. 

For the compaction, an oil-heated lab press was used with the parameters mentioned above to produce the pressed mycelium cores. The final height was adjusted using spacer discs instead of pressure. For the higher density mycelial material (550 kg/m^3^), both the pressing time and the height of the press cake were varied. The pressing temperatures and times were determined based on experience with the material; then, both a higher temperature and the pressing time have an influence on the material density. A pressing time that is too short causes insufficient compaction of the material and at the same time prevents the core temperature from being reached. A temperature that is too low leads to poor bonding of the fungal proteins with the substrate. Unpressed hemp hurds yielded a mycelial material with a density of around 135 kg/m^3^ and a thermal conductivity of 0.045 to 0.05 W/mK [36,37]. Although lower densities are theoretically possible for mycelial materials, they cannot be produced with the substrate hemp hurds [3]. After pressing, the mycelial core was trimmed and cut it into strips with appropriate pultrusion dimensions (8 mm thickness, 116 mm width and 600 mm length), making it ready for further experiments.

### 2.2. Pultrusion Process of Mycelium Material

**Figure 2:**
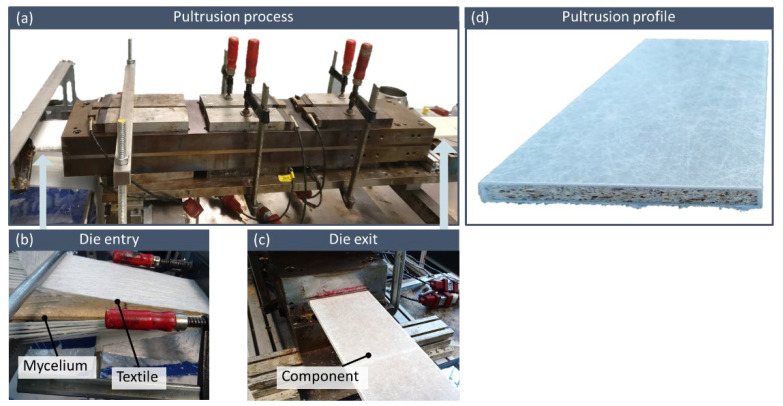
Manufacturing process (**a**) with reinforcing materials (glass fiber roving, glass fiber textiles and mycelium sandwich core) being impregnated with resin (**b**) and exiting the hot forming tool (**c**) at temperatures of up to 150 °C. The finished product is shown in full view (**d**).

In order to produce a sandwich laminate with various core materials in the pultrusion process, the authors developed a suitable textile guidance made out of metal plates which not only directs the sandwich core but also the glass fibers and textiles to the forming tool, ensuring all components are correctly positioned in the final product. The single sandwich core profiles are fed manually to the die entry, where they get pulled with the fiber reinforcements by friction. For pultrusion of the sandwich cores with a cross section measuring 8 × 116 mm², an existing die for flat profiles of 10 mm thickness and 120 mm width was chosen. The sandwich core was surrounded by unidirectional (UD) glass fibers (type PS4100, 4800 tex, European Owens Corning Fiberglas, Brussels, Belgium) and in order to enhance transverse reinforcement and improve compressibility, a glass fiber mat (type U528, 450 g/m², European Owens Corning Fiberglas, Brussels, Belgium) and a complex fabric (type VTXM 30/464/75 E05A-45/90/45 TRIAXIAL E GLASS, 574 g/m², Metyx Composites, Tuzla Istanbul, Turkey) were each used on both the top and bottom side. Figure 2 illustrates the manufacturing process. 

In this study, a conventional unsaturated polyester resin system (Norsodyne P 47127, Polynt Composites Germany GmbH, Miehlen, Germany) was employed as the matrix material, which is commonly used in pultrusion and easy to process compared to other resin systems such as epoxy or polyurethane. Two additional core materials, namely PUR foam (Gaugler & Lutz, Aalen-Ebnat, Germany) and commercially available chipboard, were chosen and analyzed next to the mycelium core. PUR foam was chosen as it is commonly used in structural applications, and chipboard was chosen because of its similar composition to mycelium. Due to different availability on the market, it was not possible to use core materials with the same density. To ensure a solid comparison in terms of lightweight potential, the densities of the core materials were chosen to be as similar as possible in a range between 100 and 650 kg/m³.

### 2.3. Material Tests

To assess the suitability of the mycelium material for sandwich constructions, basic mechanical properties of the pultruded samples were determined through a number of mechanical material tests. Additionally, comparative materials such as PUR foam and chipboard underwent identical tests. The sandwich composite’s behavior under bending load was evaluated via a 4-point bending test based on DIN 53293. Samples with a thickness of 10 mm were tested. Additional adhesion tests according to DIN 53292 were carried out to investigate the tensile strength and elasticity of the material. Therefore, samples with an area of 50 × 50 mm² were cut out of the pultruded test components. The test was performed on a Zwick UPM 50kN testing machine at 22.7 °C.

### 2.4. Simulation

**Figure 3:**
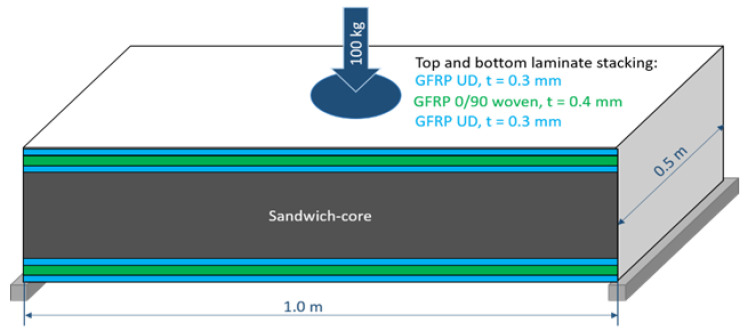
Schematic drawing of the sandwich floor panel, not to scale.

Based on the material behavior observed in the structural tests of all three sandwich materials (mycelium core, PUR foam and chipboard), a comparing design study was carried out. The study is intended to demonstrate the consequences of the differences in mechanical properties and densities of the core materials under bending loads. The study evaluates the design of a fictional floor panel, as shown in Figure 3. Similar designs could be applied, e.g., in buildings, vehicles or ships.

A length of 1.0 m, a width of 0.5 m and a centrally applied surface load of 100 kg (981 N) is assumed, which corresponds approximately to the weight of a person including clothing and equipment. The panel rests on a substructure on the left and right and is therefore subject to transverse force bending over a free-span length of 1 m. Variation of the free-span length results in a different bending moment, but is not the subject of the study. The top layers are each designed as 1 mm thick laminates of glass fiber-reinforced plastic (GFRP) consisting of a 0.4 mm thick layer of a canvas fabric and two 0.3 mm thick unidirectional (UD) layers. 

The simulation model was built in Ansys Parametric design language (APDL, Ansys Inc., Canonsburg, PA, USA)., using thick SHELL181 elements that can represent bending and shear deformations of the floor panel. The relevant elasticity values of the materials are summarized in Table 2. 

Homogenized mechanical properties of the GFRP face sheets were determined with Ansys Material Designer (Ansys Inc., Canonsburg, Pennsylvania, United States). They are calculated from the elastic behavior of a representative volume element (RVE) with a user-defined textile structure, i.e., unidirectional (UD) and 0°/90° woven. The face sheets are assumed to consist of an epoxy matrix and E-glass fiber. Young’s moduli for the mycelium, chipboard and PUR foam sandwich materials were estimated based on adhesion tests through the linear regression of the measured stress–strain curves. Experimental values of the Poisson’s ratio are not available for the core materials. The Poisson’s ratio is assumed to be 0.2 for all core materials, which corresponds to a well-compressible material. The material behavior was assumed to be isotropic. 

### 2.5. Life Cycle Assessment

The life cycle assessment (LCA) is an established method to analyze and evaluate the environmental impact of products, among other things, on the basis of ISO 14040 and ISO 14044. An LCA usually includes all environmental impacts during the production, use phase and disposal or recycling of a product, as well as the associated upstream and downstream processes (e.g., production of raw materials, consumables and supplies). After the definition of the goal and scope of the LCA, all resources are extracted and the released emissions are determined (life cycle inventory). With the help of characterization factors, the corresponding resources and emissions are then aggregated to different environmental impacts.

The results of life cycle assessments are generally used by researchers, developers and companies for, among other things, information about environmental performance, optimization potentials in the value chain and product development in an environmentally friendly way, as well as communication and certification with reliable information to partners and customers.

The goal of this study is the assessment of the environmental potential of a pultruded mycelium sandwich profile compared to commercially available fossil- but also bio-based sandwich cores. In order to ensure a broad applicability of the results, the LCA refers to a defined production volume. In detail, the production of a sandwich profile has a length of one meter and a cross-section of 10 × 120 mm², with the core measuring 8 × 116 mm². The mycelium core with a density of 350 kg/m³ was compared to a PUR foam with 200 kg/m³; the mycelium core with a density of 550 kg/m^3^ was compared to a chipboard, which has a density of 650 kg/m^3^. Due to the different densities, the production of the functional unit (one meter of sandwich profile) results in a varying component weight in each case. The mechanical properties were also not directly taken into account in the functional unit. Depending on the selected target application and functional unit, the results may therefore vary in future projects.

For the evaluation, the European Commission’s Environmental Footprint (EF 3.0) was chosen as the characterization method. However, as the most relevant impact category, the contribution to climate change in the form of the Global Warming Potential (GWP) was analyzed in detail. The material and energy flows were modelled using the software “LCA for Experts” (formerly, GaBi Software, Stuttgart, Germany) from the manufacturer Sphera^®^ (Chicago, IL, USA). LCA for Experts is an established software for the implementation of LCA in industry [38]. Next to the interface to model the material and energy flows of a technical foreground system [39], it contains high-quality databases with a variety of life cycle inventory (LCI) data as well as methods for impact assessment, such as the EF3.0 selected for this study. For the technical foreground system, primary data already measured in previous projects served as the input [39,40,41]. This includes the processing steps of mycelium deactivation and compaction as well as pultrusion [41]. For the technical background system, life cycle inventories for materials or intermediate products such as the chipboard, PUR foam, glass fiber and resin system from the GaBi Professional database version 2022.1 could be used. For the energy supply, the LCI data set for the German electricity grid mix was taken. To model mycelium cultivation, experimental data, as described in Section 2.1, are used. In detail, the considered mycelium grows on hemp hurds under ambient conditions and at room temperature. Consequently, no external electrical or thermal energy is needed to drive the process. Due to the natural humidity of the hemp hurds of 8 to 13%, no additional water is needed for mycelial growth. Around 100% of the substrate remains in the material, with 80% as hurds; 20% is converted into mycelium. Thus, a mycelium-shive composite material is formed during growth. It is assumed that the amount of biogenic CO_2_ bound in hemp hurds, approximately 1.58 kg CO_2-_eq. per kg hemp hurds, is bound in the product until a potential end of life. 

## 3. Results 

### 3.1. Material Performance 

Mycelium can be used as a core material in sandwich profiles; however, the experiments reveal that a constant thickness of the sandwich core is essential. A tolerance of maximum ±0.1 mm is necessary for a core thickness of 8 mm. When the core is too thin, material deposits on the profile can occur due to inadequate form filling. Conversely, local over-pressurization occurs, causing the core to collapse or the profile to become stuck in the molding tool. It is demonstrated that both the chipboard and the mycelium are robust enough for the pultrusion process. There is no failure of the core material during pultrusion if the manufacturing process follows the given process frame. On the other hand, the collapse of the core is noticeable immediately after pultrusion with PUR foam with a density below 200 kg/m³.

**Figure 4:**
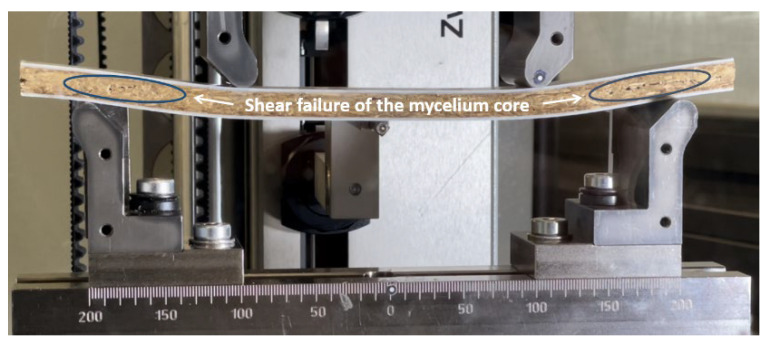
Mycelium-glass fiber reinforced polymer (GFRP) sandwich composite in 4-point bending test.

The 4-point bending test provided information about the basic elastic material behavior of the sandwich composite under bending stress. Possible modes of failure of the sandwich composites include failure of the core material, the bonding between the core and face sheets, and failure of the face sheets. Figure 4 shows a mycelium-GFRP sample in the state of core failure during the 4-point bending test. 

Cracks in the core are clearly visible in the outer area of the sample. In the central region between the load applicators, the main form of stress is pure bending. However, in the outer region of the composite material, transverse forces generate shear stress. Therefore, a shear failure of the core material can be assumed. It is noticeable that the propagation of the crack in the mycelium takes place over a long period of time at an almost constant load. Even before damage occurs, it is noticeable that the sandwich cross-sections in the outer, shear-stressed part are no longer flat and perpendicular to the neutral axis. This indicates a significant shear deformation of the beam, which is superimposed on the deflection.

**Figure 5:**
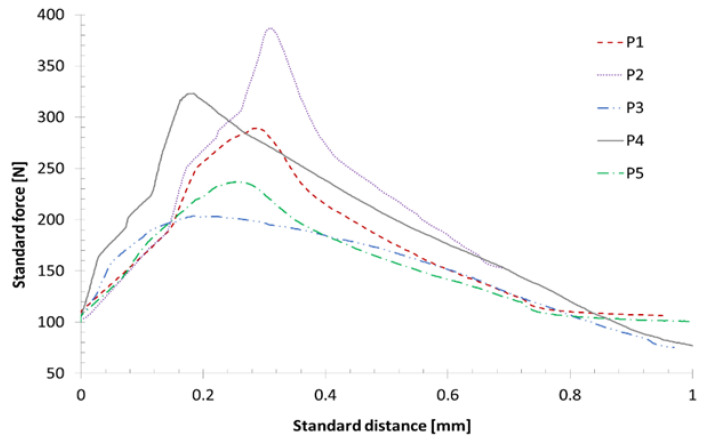
Force-displacement diagram of the frontal pull test of the mycelium samples P1–P5.

Overall, substantial differences in bending stiffness and failure load of the sandwich are detected for different core materials. Thus, the core material proves to be critical for the overall composite in terms of both stiffness and strength, which is why further selected investigations of the mechanical parameters of mycelium were conducted. Additional transverse tensile tests were carried out in accordance with DIN 53292 in order to examine the tensile strength and elasticity of the material. The force-displacement curves for five samples (P1 to P5) from the first batch of mycelial pultrudate are shown in Figure 5.

Overall, only partially elastic behavior could be observed. At the beginning of the test, settling processes in the apparatus resulted in reduced stiffness. In addition, material damage occurred before reaching the maximum force; thus, linear elastic material behavior is not present. Furthermore, there is a large variation between the individual samples. Variations likely result from the inhomogeneous material structure of the mycelium core. It is reasonable to assume that stiffness and strength can be increased by improving the homogeneity of the mycelium in production.

**Figure 6:**
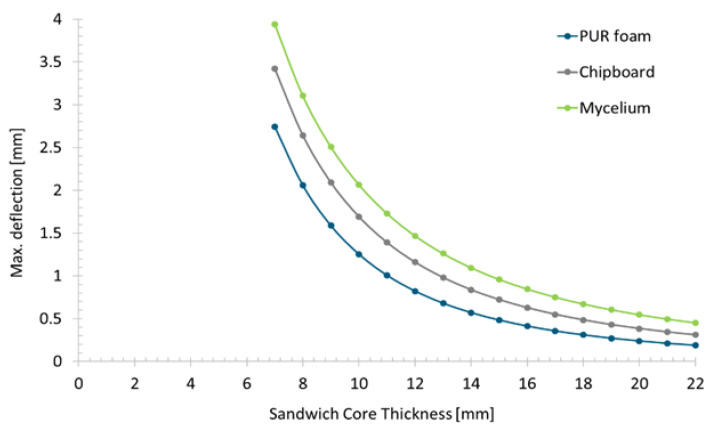
Maximum deflection in the simulation model depending on the core material and the core thickness.

In order to conduct design studies, the elasticity modulus of the material is estimated to be around 2.8 GPa from the force-displacement diagram. The average fracture stress is assumed to be around 0.1 MPa. Simulation series were carried out for the three core materials for the floor plate, with the core thickness varying between 7 and 22 mm. In general, the thicker the sandwich core, the higher the geometric bending stiffness of the overall composite and the lower the deflection. Additionally, there is a shear deformation that is significantly dependent on the stiffness of the core material. The results of the simulation series are shown in Figure 6, with the maximum deflection plotted against the thickness of the sandwich core.

For the same core thickness, the deformation is greatest for the mycelium core, as it has the lowest elasticity and shear modulus. A thicker core is required for the mycelium core to achieve the same bending stiffness as the other materials. For example, if the maximum deflection is to be limited to 1 mm, this can be achieved equally well with a PUR foam core of 11 mm thickness, a chipboard core of 13 mm thickness or a mycelium core of 15 mm thickness.

**Figure 7:**
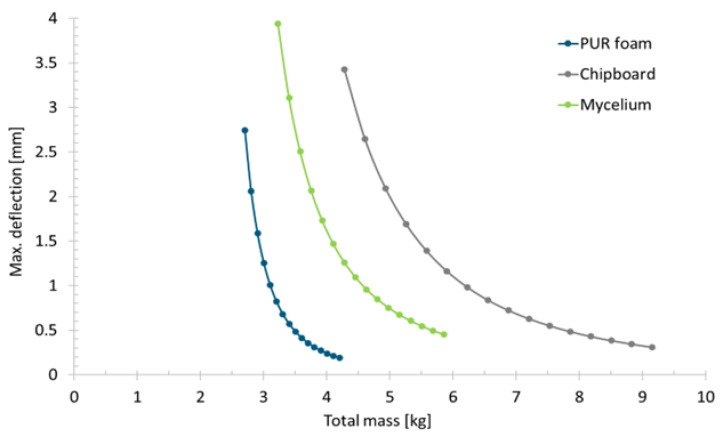
Maximum deflection in the simulation model compared to the total mass of the sandwich composite.

To assess the lightweight quality of the materials, the total weight of the sandwich composite was calculated from the thickness and density of the sandwich cores and the mass of the cover layers, as shown in Figure 7.

Due to the lower density of the mycelium core, a different order can be observed here: for a given mass of the sandwich composite, PUR foam offers the highest stiffness, followed by mycelium and chipboard. If the deflection is to be limited to 1 mm, the weight is around 3.1 kg (PUR foam), 4.6 kg (mycelium) or 6.2 kg (chipboard). For stiffness-driven lightweight construction, PUR foam seems to be the most suitable. Overall, results demonstrate that in the investigated scenario, floor panels with lower weight compared to those made of chipboard can be realized using mycelium as the core material. However, this only applies if the bending stiffness of the panel is the relevant design criterion. Due to the significantly reduced strength of mycelium compared to chipboard, different results can be expected for a strength-driven design.

### 3.2. Sustainability Potential

Regardless of the mechanical performance, a defined pultruded volume was used for an initial comparison with conventional sandwich materials. This ensured that the processing parameters were comparable in the LCA. However, due to the available density of conventional sandwich cores, no direct mass-based comparison was possible (compare Section 2.5). It should therefore be taken into account that the results only serve as a first indicator. According to the application, a function-based comparison must be carried out.

**Figure 8:**
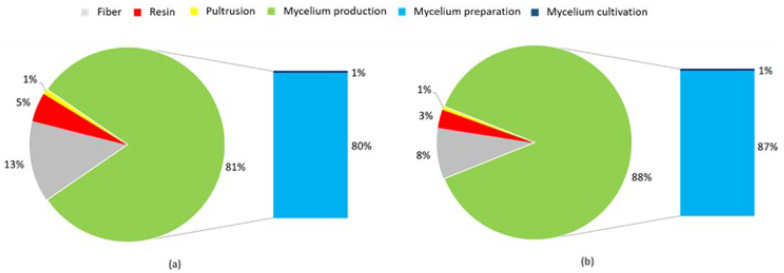
Percentage composition of the GWP for the production of the pultruded profile (dimensions 1000 × 120 × 10 mm³) with mycelium core ((**a**): density of 350 kg/m^3^ and (**b**): density of 550 kg/m^3^).

In a first step, the main drivers regarding GWP were analyzed to derive possible optimization potentials for processing. Figure 8 illustrates the share on the GWP along the process chain from the mycelium cultivation to pultrusion process for sandwich profiles with a mycelium core of densities 350 kg/m^3^ (a) and 550 kg/m^3^ (b).

Initial results demonstrate that only 1% of the GWP is caused by the pultrusion process itself (shown in yellow). The production of fibers (in gray) contributes up to 13% of the GWP, while the production of resin (in red) contributes up to 5%. The majority of emissions, up to 88%, are generated during mycelium production (shown in green), which includes both mycelium cultivation and processing, with mycelium processing being almost entirely responsible for emissions in this area. This is due to the time- and energy-intensive pressing process (180 °C for 20 to 35 min) carried out at high temperatures for compacting and sterilizing mycelium at the laboratory scale. This also explains why mycelium production for a core with 550 kg/m³ leads to a higher share of the total GWP. Deactivation and pressing require more time (see Table 1); the pultrusion process and the required structural materials, on the other hand, are independent of the sandwich density. Therefore, the share of mycelium production increases.

**Figure 9:**
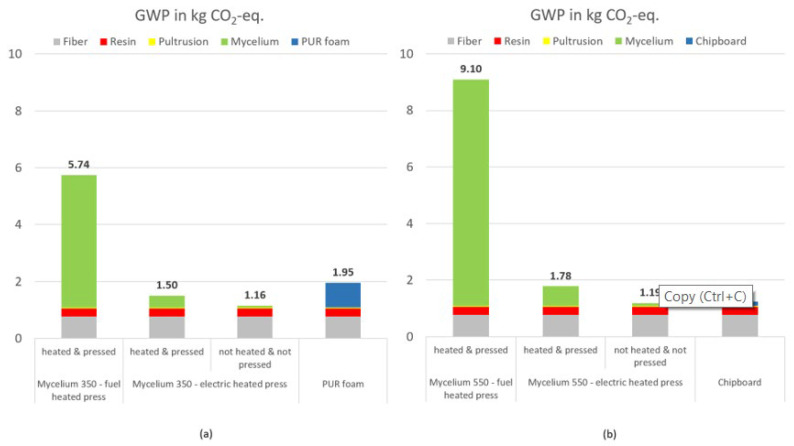
Global warming potential (GWP) for the production of a pultruded profile (dimensions 1000 × 120 × 10 mm³). (**a**) In comparison, profile with mycelium core (density 350 kg/m^3^ and component weight: 0.87 kg) taking into account various scenarios with PUR foam core (density 200 kg/m^3^ and component weight: 0.64 kg). (**b**) Comparing profile with mycelium core (density 550 kg/m^3^ and component weight 1.06 kg) considering various scenarios with chipboard core (density 650 kg/m^3^ and component weight: 1.20 kg).

However, the results were carried out on a laboratory scale; in a series application, more energy-efficient heating presses such as electrically heated presses would be used. In addition, the potential of conceivable process improvements was investigated. The aim was to identify whether these lead to a significant improvement in GWP and should therefore be addressed in future research activities. For example, due to the temperatures of up to 150 °C, the deactivation could take place directly during pultrusion. Thus, only compaction at room temperature has to be conducted in the press, which leads to both shorter process times and lower energy demand. To investigate the different potentials, the scenarios “heated and pressed” and “unheated and unpressed” was considered based on production with an electrically heated press. The latter scenario excludes both the influence of heating energy for sterilizing the mycelium and the pressing process. This is based on the assumption that deactivation can be conducted in situ during the pultrusion, and as a result, the pressing time can be significantly reduced. In the following, Figure 9 shows the GWP of sandwich profiles with mycelium core considering the scenarios with their respective comparative products.

The production of pultruded profiles with mycelium cores of densities 350 and 550 kg/m³ using an oil-heated press results in a GWP of 5.74 and 9.10 kg CO_2_-eq. on a laboratory scale. For a profile with a mycelium core density of 350 kg/m³, this corresponds to a multiplication of the GWP by a factor of 2.8 compared to the reference profile (PUR foam core). A mycelium core with a density of 550 kg/m³ leads to a 7.3-fold increase in GWP compared to the reference product, a chipboard core, mainly due to the energy-intensive mycelium processing. When the mycelium cores are processed in an electrically heated press, the GWP decreases depending on the density of the core to 1.50 kg and 1.78 kg CO_2_-eq. (350 and 550 kg/m³, respectively). For the production of the profile with a mycelium core density of 350 kg/m³, 23% fewer emissions are generated than for the reference profile with the PUR foam core. In contrast, the product with a mycelium core density of 550 kg/m³ performs 1.4 times worse than the corresponding reference product with higher emissions. Considering the “unheated and unpressed” scenario, in which the heating and pressing processes are neglected along with the entire mycelium processing, the production of profiles with mycelium cores (350 kg/m³) emits 41% less emissions, and with mycelium cores (550 kg/m³), 5% less emissions are emitted than the reference profiles.

This is highly promising given that the production of PUR foams and particleboards is highly industrialized and optimized compared to mycelium production. Further research activities, which are discussed in the next section, are necessary to improve the mycelium production processes and to replace conventionally established materials with sustainable alternatives on an industrial scale.

## 4. Discussion

The results have demonstrated that the use of mycelium as a sandwich material can lead to an improvement in the greenhouse potential not only compared to fossil-based but also bio-based conventional materials. Especially compared to the latter, the use of mycelium has further advantages, e.g., with regard to land use and water consumption, since it is mainly grown from agricultural waste with no significant water demand. However, in order to raise research from the laboratory scale to industrialization, the product design and manufacturing process chains must be holistically optimized. Especially with regard to product and process design, investigations should be expanded by further research on the use of living mycelium in the pultrusion process.

For example, the possibility of tailored growth of mycelium in the process could enable a continuous mycelium panel production. In the mycelial cores produced in the project, no additional binder is added and only the protein of the mycelium itself is used for bonding. The use of supporting natural fibers or a bio-based (binder) matrix might significantly increase or improve the mechanical strength.

Further added value can be achieved by improving the mycelium cultivation process. In this work, the production of the mycelial nuclei was carried out under controlled growing conditions. But it is to note that the mycelial material can also grow continuously outside a standard climate with only slightly more growing time (approximately, an additional 1 to 2 days). However, industrial growth production ideally uses growth times lower than 10 to 14 days, as required in this work. Therefore, the investigation of significantly more efficient biologically optimized fungal species and fungal/substrate combinations appear to be of added value.

Moreover, further investigation in the perspective of scaling up the process of deactivating and drying the mycelium material might be an interesting aspect. By using other fungus/substrate combinations, the pressing temperature could be reduced, thus saving energy during production. Trials with low pressing times have already been successfully carried out. Energy savings and higher cycle times can be assumed for a continuous pressing process. In addition, an inline deactivation in the pultrusion process can further reduce the energy consumption and improve the global warming potential.

Furthermore, in particular, a well-founded economic analysis is necessary, since the profitability will be decisive for the feasibility of scaling in an industrial context. However, an economic estimate was not the focus of this study, and a rough estimation is not possible due to the laboratory scale and the necessary research activities discussed for the continuous, industrial-scale production.

Based on the material properties, application areas are found in the automotive, railway companies, construction, mechanical engineering, or wood-based panel industries. The high energy absorption in the bending test also suggests an applicability for crash and impact endangered components. In the long term, the use of pultruded glass fiber-reinforced plastic (GFRP) sandwich profiles with a mycelium core for secondary structures in the field of eco-efficient aviation is also an interesting aspect to be investigated.

## 5. Conclusions

The use of bio-based materials such as mycelium as a substitute for fossil-based materials in processes such as pultrusion has great potential, which lies in their ability to promote resource efficiency, circular economy and sustainability. Following UN sustainability goals, they offer the opportunity to reduce dependence on finite resources, decrease the carbon footprint and develop new ecological solutions.

This study gave first insights into the potential mycelium could unfold being used as an alternative core material in pultruded sandwich profiles (on a laboratory scale) regarding performance and ecological sustainability. Looking at the environmental impact, it is noteworthy that 88% of greenhouse gas emissions are attributed to mycelium production caused by the heat pressing applied for mycelium deactivation. By replacing the oil-heated lab press with an electrically heated press, the GWP decreases from 5.74 and 9.10 kg CO_2_-eq. to 1.50 and 1.78 kg CO_2_-eq. for pultruded profiles with mycelium cores of 350 and 550 kg/m³, respectively. Additionally, first material tests demonstrated that the performance of pultruded sandwich profiles with a mycelium core appear to be competitive compared to alternative core materials such as PUR foam and chipboard, as examined in this study. To further enhance the performance of the mycelium core, it is crucial to maintain a consistent sandwich thickness.

Through continuous research and innovation along the value chain from mycelium cultivation to part manufacturing, these materials can be further developed in terms of material properties and energy efficient processing of the cores. Provided with the same volume/weight specific functional properties compared to conventional fossil-based cores, mycelium sandwich cores can drive sustainable and future-oriented development and be used in various industries such as construction, packaging and automotive sectors. In addition to improving mechanical properties and reducing energy demand during processing, further investigations, for example, towards the combination of bio-based resin and fiber systems for pultruded sandwich profiles with mycelium cores, elevate the sustainability potential of the studied composite to the next level. Additionally, through further testing involving the use of living mycelium, additional potentials towards industrialization can be unlocked.

## Figures and Tables

**Table 1:** Parameter sets for the mycelium samples.

Target Density [kg/m^3^]	Presscake Height [cm]	Pressing Temperature [°C]	Final Height [mm]	Pressing Duration [min]
300	2.75	180	8	20
550	3.5	180	8	30

**Table 2:** Elasticity characteristics of composite materials.

	$E_{1}\;[\mathrm{GPa}]$	$E_{2}\;[\mathrm{GPa}]$	$E_{3}\;[\mathrm{GPa}]$	$G_{12}\;[\mathrm{GPa}]$	$G_{13}\;[\mathrm{GPa}]$	$G_{23}\;[\mathrm{GPa}]$	$\upsilon_{12}\;[-]$	$\upsilon_{13}\;[-]$	$\upsilon_{23}\;[-]$	$\rho \;\left[\frac{kg}{m^{3}}\right]$
GFRP UD *(65% fiber volume content)*	48.8	15.1	15.1	5.5	5.5	5.5	0.25	0.25	0.38	2096
GFRP 0/90 woven *(60% fiber volume content)*	25.4	25.4	21.5	5.9	4.4	4.4	0.2	0.22	0.22	1868
Mycelium 350 kg/m³	2.8	2.8	2.8	1.0	1.0	1.0	0.2	0.2	0.2	350
Chipboard 650 kg/m³	8.5	8.5	8.5	3.0	3.0	3.0	0.2	0.2	0.2	650
PUR foam 200 kg/m³	21.5	21.5	21.5	7.7	7.7	7.7	0.2	0.2	0.2	200

## Data Availability

Data are contained within this article.
